# Special Issue: Smart Nanomaterials for Environmental Remediation

**DOI:** 10.3390/ma14010143

**Published:** 2020-12-31

**Authors:** Won San Choi

**Affiliations:** Department of Chemical and Biological Engineering, Hanbat National University, 125 Dongseodaero, Yuseong-gu, Daejeon 305-719, Korea; choiws@hanbat.ac.kr

Nanomaterials that can be reversibly or irreversibly changed in structures and properties by the influence of external chemical and physical stimuli are defined as smart nanomaterials. External chemical and physical stimuli can include pH, temperature, moisture, force, electric fields, and magnetic fields. Smart nanomaterials have been used for environmental remediation, energy generation, 3D printing, smart textiles, self-healing, and healthcare. Since oily wastewater, wastewater, and polluted air result in serious health and environmental problems, significant interest has been focused on environmental remediation using smart nanomaterials. The aim of environmental remediation using smart nanomaterials is to remove contaminants from environmental media such as groundwater, surface water, sea water soil, or air.

This Special Issue deals with the recent progresses in smart nanomaterials and related nanomaterials for environmental remediation. Our Special Issue is composed of five main topics, such as oil/water separators [1,2], adsorbents/absorbents [3,4,5], catalysts [6,7], and fundamental studies [8,9] for environmental remediation (Figure 1). Herein, I summarize each topic discussed in our Special Issue. This editorial begins with oil/water separators for the separation of water or oil in oil or water, respectively. Then, adsorbents/absorbents, air filters, and catalysts are presented. Finally, fundamental studies for environmental remediation are discussed.

Recently, oily wastewater has become a serious environmental problem and threat. To address this issue, various synthetic methods and nanomaterials have been proposed for oil/water separation [1]. Since the wettability and functionality of oil/water separators can be controlled by using nanomaterials, nanomaterials have been coated or incorporated onto/into the superhydrophobic or superhydrophilic oil/water separator. Oil/water separators loaded with nanomaterials can perform versatile missions. They can eliminate heavy metal ions or organic pollutants in water using an adsorption process during the oil/water separation. They also remove organic pollutants in water using catalytic decomposition. However, nanomaterials could be leaked into the environment because it is very difficult to monitor the leakage of nanomaterials due to their extremely small size, resulting in a severe threat to the environment and public health.

To solve the abovementioned problems, Choi et al. propose using environmentally friendly materials that are part of nature and nontoxic to nature for superhydrophobic and superhydrophilic oil/water separators [2]. Superhydrophobic (MFS/CC-PDMS) and superhydrophilic (MFS/CC-DKGM) sponges are prepared by a simple coating of pristine sponge (melamine formaldehyde sponge, MFS) with a mixture of calcium carbonate (CC) rods and deacetylized Konjac glucomannan (DKGM) (or polydimethylsiloxane (PDMS)). These superhydrophobic and superhydrophilic oil/water separators can be used as absorbents and filters. The superhydrophobic sponge absorbent selectively absorbed only oils from various types of oil/water mixtures. Their absorption capacity reached 52–76 g/g. They were also used as filters for oil/water separation. Before filtration, the superhydrophilic and superhydrophobic filters are prewetted with water and oil, respectively. Only water and oil rapidly penetrate the superhydrophilic and superhydrophobic filters, respectively, when the oil/water mixture is poured. The superhydrophilic and superhydrophobic filters showed excellent water- and oil-flux performances of 4702 L/m^2^ h and 19,591 L/m^2^ h, respectively. Furthermore, the superhydrophobic/superhydrophilic absorbents or filters maintained their wettability characteristics relatively well even after the mechanical, chemical, and thermal stimuli.

Random discharges of organic pollutants and heavy metal ions have become a significant environmental issue in developing countries. These pollutants in groundwater and seawater endanger the eco-system and human health due to their poisonous characteristics. Heavy metal ions can be accumulated in living organisms through the food chain and remain for a long time because of their non-biodegradable characteristics. Thus, the purification of water contaminated by organic pollutants and heavy metal ions is vital to the eco-system and public health. Numerous techniques including adsorption, ion-exchange, reverse osmosis, bio-sorption, and precipitation have been proposed for the purification of contaminated water. Among the abovementioned methods, adsorption and ion-exchange techniques are widely used due to their high efficiency, simplicity, and low cost.

Adsorbents are generally used for the removal of heavy metal ions and organic pollutants in wastewater. The removal mechanism includes electrostatic interaction, ion-exchange reaction, coordination reaction, and pi-pi interaction. Macario et al. report a water treatment method for industrial waste using the ion exchange technique [3]. Heavy metals in the water can be significantly removed by ion exchanger, ETS-10. The ETS-10 showed a better removal efficiency than commercial zeolite A in a short reaction time (30 min) for the removal of manganese, zinc, and lead. However, the removal efficiencies of ETA-10 and zeolite A were similar for manganese, calcium, and iron. Since the removal efficiency is affected by the hydrated radius of the ions, lead (II) possessing the lowest hydrated radius can be rapidly and completely eliminated by the ETS-10 ion exchanger. Since the ETS-10 possesses high removal efficiency, stability, economy, and regeneration compared to other ion exchange materials, the ETS-10 could be one of the potentially competitive materials.

Pham et al. report the synthesis of alumina nanoparticles (NPs) by a solvothermal method for the removal of Rhodamine B in wastewater [4]. They used an anionic surfactant such as a sodium dodecyl sulfate (SDS) to enhance the surface charge of alumina NPs. The SDS-coated alumina NPs can capture cationic dye, Rhodamine B, using electrostatic interaction. The SDS-coated alumina NPs showed excellent removal efficiency and adsorption capacity (165 mg/g) for Rhodamine B. The removal efficiency of the SDS-coated alumina NPs was higher than 86% even after the 4th adsorption reaction.

Absorbents have been used intensively for the removal of organic solvents and oils because of their high absorption capacity and convenient use. Thus, most studies have focused on developing hydrophobic absorbents. No attention has been paid to hydrophilic absorbents that can eliminate strong acid and base solutions. Choi et al. report a novel absorbent for the removal of strong acid and base solutions [5]. Needlelike iron oxide-coated sponge (NIOS) absorbents with various IO layer thicknesses were prepared. NIOS absorbents quickly absorbed a strong base solution (50% m/m) in 0.5 s, with a high absorption capacity of 34,000% in the absence of an additional external force. The NIOS absorbents could also absorb a strong acid solution (15%, v/v) in 0.31 s with a high absorption capacity of 10,810% without overflow and absorption delay. The NIOS absorbents generated fewer particulate matters than the level of a class 100-clean room. The NIOS absorbents showed high absorption capacity and speed because they possessed a needlelike surface morphology inducing a capillary force. By these reasons, the NIOS absorbents could prevent the solution overflow and formation of droplet fragments during the removal of strong acid and base solutions.

A significant amount of organic pollutants has been generated annually by the dyeing and textile industries. If organic pollutants are discharged without proper treatment, organic pollutants will contaminate and endanger water resources and living creatures. These organic pollutants consist of benzene ring compounds such as Rhodamine B, Rhodamine 6G, methylene blue, methyl orange, and Congo red. Thus, they are seldom biodegraded in nature. Various treatment approaches have been developed, including adsorption, catalytic decomposition, biological degradation, and so on. Of these methods, catalytic decomposition is an effective method for the decomposition of organic pollutants. 

Liao et al. report a catalytic decomposition of Rhodamine 6G using iron NP-coated montmorillonite [6]. To overcome the passivation and aggregation of iron NPs, the iron NP-coated montmorillonite was synthesized by the coating of iron NPs on montmorillonite. They used the Fenton reaction of iron NP-coated montmorillonite for catalytic decomposition of Rhodamine 6G under microwave irradiation. With the assistance of microwave irradiation, the Fenton reaction of iron NP-coated montmorillonite can induce the formation of hydroxyl radicals (•OH) that can decompose the organic pollutant such as the Rhodamine 6G. The iron NP-coated montmorillonite exhibited a high removal amount and a fast removal rate of 0.5 g/g and 0.46 /min, compared to previously reported values.

Wei et al. report the synthesis of Ag_2_S quantum dot-coated SnS_2_ (Ag_2_S@SnS_2_) nanostructures for the photocatalytic decomposition of Rhodamine B [7]. The Ag_2_S quantum dots were coated onto the SnS_2_ nanostructures to form a heterojunction between Ag_2_S and SnS_2_ via in-situ ion exchange reaction, which led to the enhancement of catalytic activity of Ag_2_S@SnS_2_ photocatalysts. The Ag_2_S@SnS_2_ photocatalysts showed excellent catalytic performance with good cycling stability, compared to other catalysts. Organic pollutants, such as Rhodamine B, can be decomposed by active species such as hydroxyl radicals and photogenerated holes. The decomposition efficiency of Rhodamine B reached 96.6% under the simulated sunlight irradiation. 

Finally, our Special Issue also pays attention to the fundamental studies for environmental remediation. A deep understanding of theory, mechanism, synthesis, and measurement is crucial for applications of the abovementioned topics. 

Koo et al. study the effect of potassium ions on the formation of manganese oxide/graphene nanocomposites [8]. Mixed-valence manganese oxide (MnO_x_) was synthesized onto the potassium (K) ion-doped reduced graphene oxide (rGO) composites. For the synthesis of the MnO_x_, two types of precursors including potassium permanganate and manganese nitrate were used. MnO_x_/rGO composites with a high specific capacitance of 1955.6 F/g were achieved by directly annealing the rGO in the absence of a purification step. The mixed-valence MnO_x_ can be stably formed on the surface of the rGO by the assistance of potassium ions. This study discussed the synthesis of metal oxide NPs-coated graphene and the stabilization/dispersion of NPs on the graphene, which could be applied to the adsorption and catalytic reaction of organic pollutants.

Kameyama et al. study the water-retention properties of biochar prepared at different pyrolysis conditions [9]. Since the properties of biochar that are used as a soil amendment material can be different depending on law material and pyrolysis condition, they studied the effect of law materials type and pyrolysis condition on the water-retention related properties of biochar. The distribution of micrometer-sized pores of biochars was not affected by varying the pyrolysis temperature, which suggested that the pyrolysis temperature did not affect plant-available water capacities. The hydrophobicity index decreased as a function of increasing temperatures. This study discussed tuning the physicochemical properties of biochar by varying the preparation conditions and choosing law materials, which could be used for tuning the chemical and physical properties of adsorbents such as biochar.

## Figures and Tables

**Figure 1:**
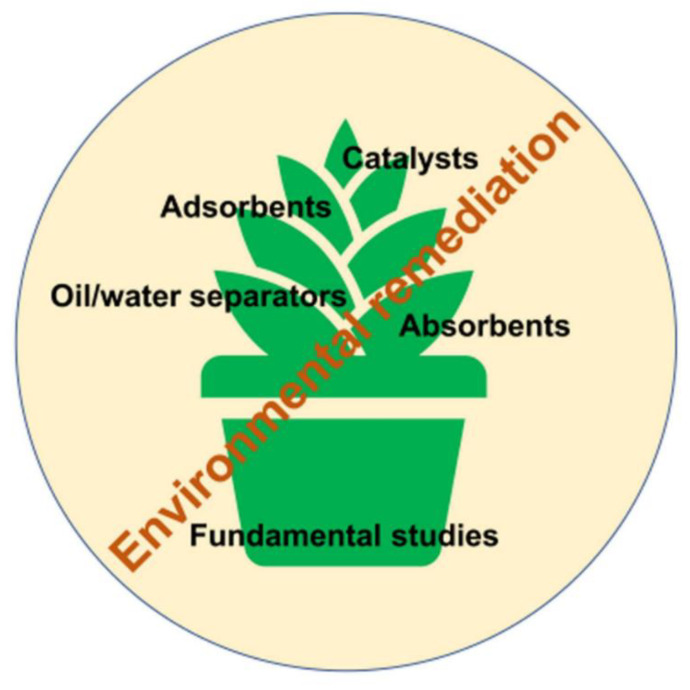
Schematic illustration of five main topics for environmental remediation.

## Data Availability

Data sharing not applicable.

## References

[1] Lee H.-J., Choi W.S. (2020). 2D and 3D Bulk Materials for Environmental Remediation: Air Filtration and Oil/Water Separation. Materials.

[2] Lee Y.S., Lim Y.K., Choi W.S. (2019). One-Step Synthesis of Environmentally Friendly Superhydrophilic and Superhydrophobic Sponges for Oil/Water Separation. Materials.

[3] Luca P.D., Bernaudo I., Elliani R., Tagarelli A., Nagy J.B., Macario A. (2018). Industrial Waste Treatment by ETS-10 Ion Exchanger Material. Materials.

[4] Chu T.P.M., Nguyen N.T., Vu T.L., Dao T.H., Dinh L.C., Nguyen H.L., Hoang T.H., Le T.S., Pham T.D. (2019). Synthesis, Characterization, and Modification of Alumina Nanoparticles for Cationic Dye Removal. Materials.

[5] Han N., Park S., Kaang B.K., Jang W., Koo H.Y., Choi W.S. (2019). An Active Absorbent for Cleanup of High-Concentration Strong Acid and Base Solutions. Materials.

[6] Rao W., Liu H., Lv G., Wang D., Liao L. (2018). Effective Degradation of Rh 6G Using Montmorillonite-Supported Nano Zero-Valent Iron under Microwave Treatment. Materials.

[7] Zhao W., Wei Z., Ma L., Liang J., Zhang X. (2019). Ag_2_S Quantum Dots Based on Flower-like SnS_2_ as Matrix and Enhanced Photocatalytic Degradation. Materials.

[8] Jang W., Jeon D.Y., Lee Y.S., Koo H.Y. (2019). Effect of Potassium Ions on the Formation of Mixed-Valence Manganese Oxide/Graphene Nanocomposites. Materials.

[9] Kameyama K., Miyamoto T., Iwata Y. (2019). The Preliminary Study of Water-Retention Related Properties of Biochar Produced from Various Feedstock at Different Pyrolysis Temperatures. Materials.

